# CRISPR‐based knock‐in mutagenesis of the pioneer transcription factor FOXA1: optimization of strategies for multi‐allelic proteins in cancer cells

**DOI:** 10.1002/2211-5463.13139

**Published:** 2021-03-20

**Authors:** Shen Li, Joseph P. Garay, Colby A. Tubbs, Hector L. Franco

**Affiliations:** ^1^ The Lineberger Comprehensive Cancer Center Department of Genetics University of North Carolina at Chapel Hill NC USA

**Keywords:** breast cancer, Cas9, CRISPR, FOXA1, knock‐in, MCF7

## Abstract

Precise genome engineering of living cells has been revolutionized by the introduction of the highly specific and easily programmable properties of the clustered regularly interspaced short palindromic repeats (CRISPR) technology. This has greatly accelerated research into human health and has facilitated the discovery of novel therapeutics. CRISPR‐Cas9 is most widely employed for its ability to inactivate or knockout specific genes, but can be also used to introduce subtle site‐specific substitutions of DNA sequences that can lead to changes in the amino acid composition of proteins. Despite the proven success of CRISPR‐based knock‐in strategies of genes in typical diploid cells (i.e., cells containing two sets of chromosomes), precise editing of cancer cells, that typically have unstable genomes and multiple copies of chromosomes, is more challenging and not adequately addressed in the literature. Herein, we detail our methodology for replacing endogenous proteins with intended knock‐in mutants in polyploid cancer cells and discuss our experimental design, screening strategy, and facile allele frequency estimation methodology. As proof of principle, we performed genome editing of specific amino acids within the pioneer transcription factor FOXA1, a critical component of estrogen and androgen receptor signaling, in MCF‐7 breast cancer cells. We confirm mutant FOXA1 protein expression and intended amino acid substitutions via western blotting and mass spectrometry. In addition, we show that mutant allele frequency estimation is easily achieved by topoisomerase‐based cloning combined with allele‐specific PCR, which we later confirmed by next‐generation RNA‐sequencing. Typically, there are 4 ‐ 5 copies (alleles) of FOXA1 in breast cancer cells, making the editing of this protein inherently challenging. As a result, most studies that focus on FOXA1 mutants rely on ectopic overexpression of FOXA1 from a plasmid. Therefore, we provide an optimized methodology for replacing endogenous wild‐type FOXA1 with precise knock‐in mutants to enable the systematic analysis of its molecular mechanisms within the appropriate physiological context.

## Graphical abstract

Herein, we detail our CRISPR‐based site‐specific knock‐in mutagenesis methodology to target multi‐allelic proteins in cancer cells. We discuss our experimental design, screening strategy, and facile allele‐frequency estimation methodology. As proof of principle, we performed genome editing of specific amino acids within the pioneer transcription factor FOXA1, a critical component of estrogen and androgen receptor signaling.

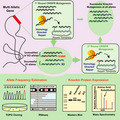

**Abbreviations**
ASP: allele‐specific PCR
Cas9: CRISPR‐associated endonuclease Cas9
CRISPR: clustered regularly interspaced short palindromic repeats
crRNAs: CRISPR RNAs
DSB: double‐stranded break
HDR: homology‐directed repair
IP: immunoprecipitation
KI: knock‐in
NHEJ: nonhomologous end joining
PAM: protospacer adjacent motif
SDM: site‐directed‐mutagenesis
sgRNA: single‐guide RNA
TOPO cloning: topoisomerase‐based cloning
tracrRNA: trans‐activating crRNA

Clustered regularly interspaced short palindromic repeats (CRISPR) and its associated components were originally discovered as an RNA‐guided adaptive immune system in bacteria and archaea [1, 2, 3]. This defense mechanism allows microorganisms to integrate short fragments of exogenous pathogenic DNA sequences into their own genome to generate a library of CRISPR RNAs (crRNAs) that serve as a memory of past genetic aggressions. Once transcribed, these crRNAs form a complex with endogenous trans‐activating crRNAs (tracrRNA) and nucleases (Cas proteins) to form active ribonucleoprotein complexes that can search and destroy foreign invading DNA sequences [3, 4, 5, 6, 7, 8]. The programmable properties of CRISPR, that originated as a natural defense mechanism in bacteria, have now been repurposed for RNA‐guided cleavage of any DNA sequence in mammalian cells [3, 9].

The type II CRISPR‐CRISPR‐associated endonuclease Cas9 (Cas9) system has been engineered for optimal efficiency in human cells. This technology combines a crRNA and tracrRNA into a single‐guide RNA (sgRNA) that can be programmed to deliver the Cas9 nuclease to any specific DNA sequence [9, 10]. Once Cas9 is bound to a target sequence, it creates a double‐stranded break (DSB) causing the activation of cellular pathways needed for DSB repair. It is at this point that researchers can edit the sequences in the vicinity of the DSB. There are two main DNA repair pathways in mammalian cells, nonhomologous end‐joining (NHEJ) repair pathway and the homology‐directed repair (HDR) pathway [11, 12, 13]. NHEJ is facile mechanism for DNA repair because it is less energetically demanding than HDR and it does not require a repair template. However, NHEJ is more error‐prone and can produce frameshift mutations that abrogate gene function. This approach allows researchers to study the consequences of the ablation of a specific DNA sequence or gene and, in most cases, creating knockout cell lines is relatively straightforward [9]. Alternatively, the DSB may be repaired by the higher fidelity HDR pathway with the aid of a repair template. This repair template can be delivered exogenously and its sequence can be designed to contain specific nucleotide substitutions that can be incorporated into the endogenous DNA sequence. If the desired nucleotide substitutions are made within the coding sequences of proteins, researchers have the ability to change the resulting amino acid composition of the target protein (Fig. 1). These site‐specific amino acid substitutions are referred to as knock‐in (KI) mutations [9, 14, 15, 16]. As compared to typical knockout experiments, the success of CRISPR‐mediated KI experiments depends on both the efficiency of DSB caused by the CRISPR‐Cas9 and the effective delivery of the repair template. Of note, some cancer cell lines have inactivated HDR pathways and can only repair DSB using the error‐prone NHEJ pathway, effectively nullifying the ability to create KIs in that cell line. These considerations make KIs considerably more challenging than knockouts. In most cases, especially in polyploid cells, this system is not entirely efficient and successfully edited KI alleles typically coexist with either wild‐type or frameshift alleles. This illustrates an inherent challenge in applying CRISPR‐based site‐specific mutagenesis to multi‐allelic genes in polyploid cancer cell lines, a scenario that is likely common and of importance to the scientific community.

**Fig. 1:**
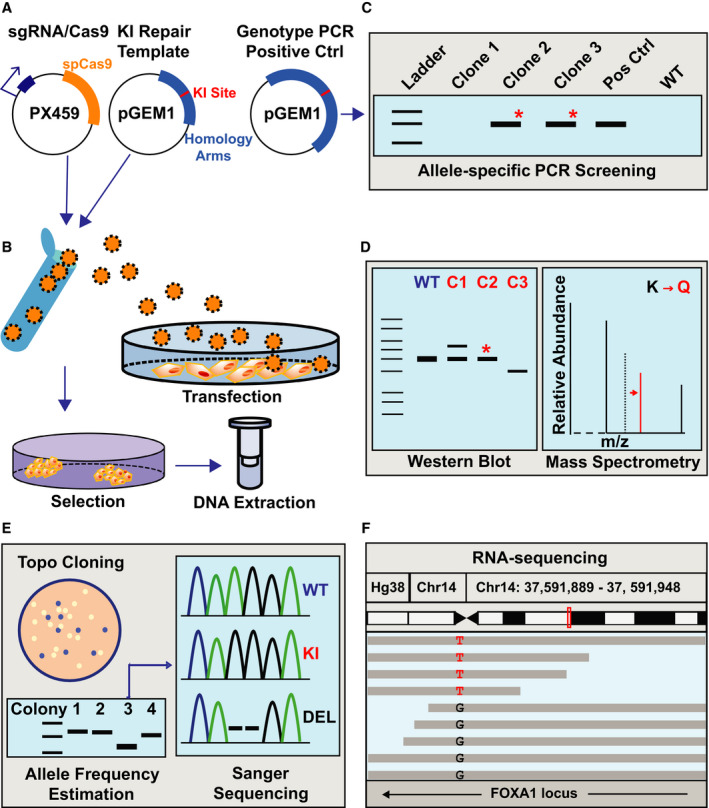
General workflow for CRISPR‐based site‐specific mutagenesis of multi‐allelic genes in cancer cell lines. (A) Three plasmids used for mutagenesis. The plasmid expressing spCas9 and the sgRNA (pX459) and the repair template plasmid containing the homology arms to the intended target site as well as the desired nucleotide substitutions for mutagenesis (pGEM1) are transfected into the cells. A third plasmid, containing a larger fragment of the endogenous locus sequence plus the desired nucleotide substitutions for mutagenesis, is used as a positive control for the ASP genotyping strategy. (B) Transfections, antibiotic selection, colony picking, and genomic DNA extraction for genotyping of potential KI clones are discussed in the text. (C) ASP is used as a screening strategy to identify clones that have successfully integrated the repair template sequence with desired nucleotide substitutions (KI). (D) Western blotting and mass spectrometry (LC‐MS/MS) is used to confirm that KI clones successfully produce full‐length KI proteins. (E, F) Allele frequency estimation is performed using TOPO cloning‐based screening of genomic DNA extracted from individual KI clones and/or the use of next‐generation sequencing approaches such as RNA‐sequencing to measure the abundance of RNA transcripts that contain the desired KI mutations. The details of each step are discussed throughout the text.

For our studies, we sought to specifically modify the pioneer transcription factor FOXA1 in the Luminal A breast cancer cell line MCF‐7. Pioneer factors are characterized for their ability to associate with condensed chromatin independently of other factors to directly modulate chromatin accessibility. FOXA1 is known as a key pioneer transcription factor for nuclear receptors such as estrogen receptor (ER) and androgen receptor (AR) in breast and prostate cancers, respectively [17, 18, 19]. FOXA1's pioneering functions stem from its protein structure that contains features of both linker histones and conventional transcription factors allowing it to displace linker histones in compacted nucleosomes. This increases chromatin accessibility and facilitates access to chromatin for other transcription factors such as ER and AR [17, 18, 19]. Thus, altering the amino acid composition of FOXA1 will alter its pioneering function across the genome and consequently alter ER and AR signaling pathways. Of note, there are multiple copies of FOXA1 in breast cancer cells (typically 4–5 alleles) making the editing of this protein inherently challenging. As a result, virtually all publications that focus on FOXA1 mutants rely on ectopic overexpression of FOXA1 from a plasmid.

CRISPR‐based mutagenesis of FOXA1 at the endogenous locus offers several advantages over the more traditional approach of ectopic overexpression from a plasmid. First, it is possible to eliminate the wild‐type version of the protein by replacing all of the copies with the intended KI variants. This provides a homogenous model system where the phenotypic outcomes are solely due to the KI variants and not due to a combination of overexpressed and wild‐type proteins typically seen with ectopic overexpression experiments. Second, editing directly within the endogenous loci will maintain the local chromatin and regulatory mechanisms intact, thus producing physiologically relevant levels of protein expression. Finally, the CRISPR‐Cas9 system is more easily implemented while introducing similar levels (or sometimes less) off‐target effects as compared to other genomic engineering techniques such as zinc finger nucleases [20, 21] and TALENs [22, 23, 24].

Herein, we have detailed our strategy for editing FOXA1 in the polyploid cancer cell line MCF‐7. We provide a reproducible protocol, screening methodology, and allele frequency estimation strategy. Verification steps and important considerations for characterizing KI cell lines are also discussed. Figure 1 illustrates the general workflow, and we have outlined our procedure in Table 1.

**Table 1:** Tasks and timeline for CRISPR‐based site‐specific mutagenesis in polyploid cancer cells.

	Task	Duration
1	Guide sgRNA design and Cas9/sgRNA plasmid construction	1 week
2	Design of the HDR template and KI screening control plasmid	2 weeks
3	Cell transfection, selection, collection, and DNA purification	8 weeks
4	Genotyping through ASP	2 weeks
5	Protein expression and mass spectrometry of KI clones	2 weeks
6	Allele frequency estimation in KI clones	2–4 weeks

## Materials and methods

### sgRNA design and Cas9/sgRNA plasmid construction

A critical step in efficient editing of mammalian cancer cell lines is the design of the guide RNA (henceforth referred to as *sgRNA*) needed to deliver Cas9 to your genomic region of interest. For this application, we used the mammalian expression vector pSpCas9(BB)‐2A‐Puro (pX459) V2.0 that transcribes the sgRNA from a U6 promoter, produces Cas9 derived from *Streptococcus* *pyogenes* (spCas9), and contains a puromycin resistance gene needed for clonal selection (Fig. 1). This plasmid was generated by Feng Zhang's laboratory and is available from the plasmid repository Addgene (plasmid ID 62988) [9].

For our proof‐of‐principle example, we focused on site‐specific amino acid substitutions of the pioneer transcription factor FOXA1 in the breast cancer cell line MCF‐7. There are 4–5 copies of FOXA1 in MCF‐7 cells depending on the strain and source of MCF‐7 cells used. We specifically targeted lysine 295 (K295) and detail the steps to mutate this lysine residue to glutamine (K295Q; Fig. 1).

There are several online resources available for sgRNA design. CRISPOR (http://crispor.org/) is an easy‐to‐use, freely available, and a well‐vetted design tool that generates several putative guide RNA target sequences along with a series of specificity, efficiency, and off‐target scores that can be used to select the best sgRNA [25]. The human genomic sequence of FOXA1 was obtained from the National Center for Biotechnology Information database (NCBI, FOXA1: NG_033028.1) and 300 bp of genomic sequence, centered on the KI site of interest (lysine 295, K295), was submitted to the CRISPOR website. sgRNA sequences with a value of 80 or higher for the MIT score and 50 or higher for the Doench score were considered good candidates. To minimize off‐target effects, sgRNAs that were predicted to have few or no off‐target sites were selected. Of note, sgRNA sequences depend on the nature of the genomic sequence at the target loci, and in some cases, high specificity scores and efficiency scores are simply not obtainable. If all possible sgRNAs have potential off‐target sites, then preference is given to sgRNAs whose off‐target sites are located in noncoding regions versus coding regions of the genome. The targeting sequence for K295 of FOXA1 is shown below. This includes the 20bp targeting guide sequence (blue font) plus 3bp for the protospacer adjacent motif (PAM) sequence (orange font). The PAM is a short sequence that must immediately follow on the 3′ end of the targeting guide sequence. These 3bp are required for proper cleavage by the Cas9 nuclease. The PAM requirement for *S. pyogenes* Cas9 nuclease is any nucleotide followed by two guanines (5′‐NGG) [9].

FOXA1 K295 targeting sequence: 5′‐AGCGGGGGCAGCGGCGCCAAGGG‐3′

To prepare oligonucleotide sequences for cloning into the pX459 plasmid, the 3′ NGG PAM sequence was deleted from the CRISPOR output sequence, and the BbsI restriction enzyme motif sequence 5′‐*CACC‐3′* was added to the 5′ end (green font) of the remaining 20 bp targeting guide sequence (blue font). Due to the nature of the U6 promoter from which the sgRNA is transcribed, a guanine (G) is added immediately 5′ of the oligonucleotide in order to allow for efficient transcription (black font) [9]. This generates the forward sgRNA oligonucleotide:

FOXA1 K295 Forward sgRNA Oligo: 5′‐CACCGAGCGGGGGCAGCGGCGCCAA‐3′

To generate the reverse strand oligo, the reverse complement was obtained from the 20 bp targeting guide sequence (blue font) and the BbsI restriction enzyme motif sequence 5′‐*AAAC‐3*′ was added to the 5′ end (green font). This completes the reverse strand sgRNA oligonucleotide:

FOXA1 K295 Reverse sgRNA Oligo: 5′‐AAACTTGGCGCCGCTGCCCCCGCTC‐3′

As with most CRISPR‐based experiments, it is always advisable to design more than one sgRNA. Especially since some sgRNAs will work better than others depending on the cell context. When attempting to edit genes with multiple alleles, more than one sgRNA is needed in the case where some, but not all, of the alleles are successfully edited. In most cases, the unedited alleles will contain small indels that will ablate the binding site or efficacy of the original sgRNA used. Therefore, a second sgRNA targeting a different location (slightly upstream or downstream) of the first sgRNA will allow for a second round of transfection to target the unedited alleles and create homogenous clones. These transfection strategies are discussed in the ‘Transfection and selection of KI cell lines’ section of this manuscript.

**Fig. 2:**
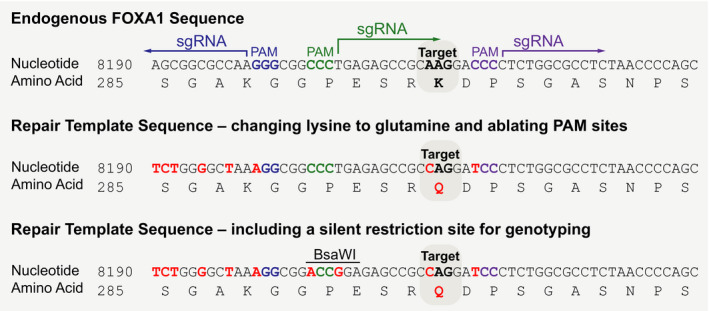
Design of the repair template sequence for precise genome editing. The human FOXA1 reference sequence is shown at the top. The target site lysine 295 (K295) is highlighted and the three sgRNAs are shown along with their associated PAM sites. To design the repair template, the desired nucleotide substitutions are made to specifically change the amino acid sequence of the target protein. In addition, several silent mutations are introduced to alter the endogenous PAM sites to avoid repeated cleavage by Cas9 (middle sequence). Silent mutations that created a restriction enzyme recognition site were also designed into the repair template to provide an alternative genotyping strategy (bottom sequence).

For FOXA1, we designed three different sgRNAs, all centered at the target amino acid (K295; Fig. 2). For efficient editing, it is recommended to design the sgRNAs at or immediately adjacent to the desired target amino acid.

FOXA1 K295 sgRNA #1 5′‐AGCGGGGGCAGCGGCGCCAA‐3′
FOXA1 K295 sgRNA #2 5′‐TGGGGTTAGAGGCGCCAGAG‐3′
FOXA1 K295 sgRNA #3 5′‐AGGGGTCCTTGCGGCTCTCA‐3′

For each of the three guide sequences, the BbsI restriction enzyme motifs were added to the 5′ and 3′ ends (as shown above) and the resulting oligonucleotides were ordered from Integrated DNA Technologies (IDT). The sgRNA oligonucleotides were resuspended in water to a final concentration of 100 μm and the corresponding forward and reverse oligos were annealed and cloned into the pX459 plasmid using the protocol described by Ran *et al*. [9]. Briefly, 1 μL of the forward sgRNA oligo (100 μm) was annealed with 1ul of the reverse sgRNA (100 μm) using 1 μL 10× T4 ligation buffer (New England Biolabs, NEB), 1 μL T4 PNK (NEB), and 6 μL of water. The annealing reaction was performed in a thermocycler using the following parameters: 37 °C for 30 min, 95 °C for 5 min, and final ramp down to 25 °C at 5 °C per minute (about 15‐min ramp down).

The annealed sgRNA oligos were diluted 1 : 200 in water and ligated into the pX459 plasmid that has been linearized with restriction enzyme BbsI (New England Biolabs). The ligation mixture contained 2 μL of diluted sgRNA oligos, 100 ng of linearized PX459 plasmid, 2 μL of 10× T4 ligation buffer, and 1 μL T4 DNA ligase (NEB). The ligation reaction was performed in a thermocycler at 37 °C for 5 min followed by 21 °C for 5 min.

The ligated plasmid was transformed into DH5α competent *Escherichia coli* cells (Invitrogen, 18265017) and spread onto LB agar plates containing 100 μg·mL^−1^ ampicillin. Plasmids were prepared using silica‐membrane‐based kit (Plasmid Miniprep Kit; Qiagen) following the manufacturer's instructions and quantified using Nanodrop Spectrophotometer. Successful integration of the sgRNA was confirmed by Sanger sequencing using the common ‘U6’ sequencing primer, 5′‐GGCCTATTTCCCATGATTCC‐3′.

### Design of the repair template sequence for precise genome editing

There are several key features of the repair template that need to be considered when designing the sequences for site‐specific mutagenesis. The repair template should include the desired nucleotide substitutions needed to specifically change the amino acid sequence of the target protein. In addition, the repair template should include silent mutations (i.e., nucleotide substitutions that do not change the amino acid sequence) designed to alter the endogenous PAM site or the endogenous 20 bp gRNA targeting sequence in order to avoid repeated cleavage by Cas9 once the cell has successfully repaired the DSB (Fig. 2). Silent mutations that create a restriction enzyme recognition site may also be designed into the repair template in order to provide an alternative genotyping strategy if desired. This must be designed in a way that does not alter the amino acid sequence of your protein or the open reading frame.

To design the repair template for modification of K295 on FOXA1, the entire genomic sequence (including the 5′ UTR, introns, and 3′UTR) was obtained using the NCBI genomic database (FOXA1: NG_033028.1). The genomic sequence was annotated to identify the start codon, open reading frame, and corresponding amino acids. The PAM recognition sites for each of the three sgRNAs are also annotated in the sequence (Fig. 2).

To generate the intended mutation, the repair template sequence was designed to contain a single nucleotide substitution needed to convert lysine at position 295 into a glutamine (AAG to CAG). In addition, silent mutations were introduced into the repair template sequence in order to ablate the PAM recognition sequence (or other significant nucleotides within the sgRNA binding site) [26]. This prevents additional rounds of cutting by Cas9 once the DSB has been successfully repaired using the provided repair template (Fig. 2). As a final step, we introduced silent mutations that created a restriction enzyme site within the target locus using the Watcut online tool (http://watcut.uwaterloo.ca/). This provides an alternative genotyping strategy (genomic PCR followed by restriction digest) to identify clones that have successfully repaired the endogenous locus using the repair template. For FOXA1, the restriction enzyme BsaW1 was used because there are no naturally occurring restriction sites within 300 bp upstream or downstream of the edited region (Fig. 2). For genotyping, primers were designed to amplify 300 bp upstream and 300 bp downstream of the target amino acid and thus limited our restriction enzyme motif search to this stretch of DNA. Genotyping strategies are discussed in more detail below.

### Preparation of the repair template plasmid and the positive control plasmid needed for allele‐specific PCR screening

**Fig. 3:**
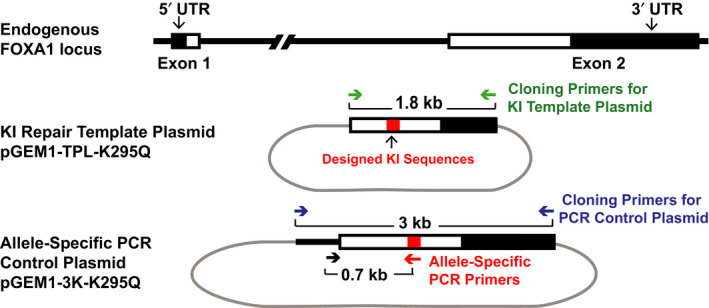
Repair template plasmid and PCR screening positive control plasmid preparation. The human FOXA1 endogenous locus is shown at the top. Two different plasmids were generated, the repair template plasmid and an ASP positive control plasmid to be used during the screening of potential KI clones. The repair template plasmid was built on the pGEM1 backbone and contains the FOXA1 repair template sequence (shown in Fig. 2) along with FOXA1 homology arms of 600 bp upstream and 1200 bp downstream of K295. The ASP ‐positive control plasmid was intentionally designed to contain more of the endogenous sequence of FOXA1 as well as the repair template sequence to be able to test our ASP primers. The forward primer only hybridizes to this plasmid and not the repair template plasmid thus allowing us to detect successful KI editing within the endogenous locus of FOXA1 and not random integration of the repair plasmid.

Once the sequence for the repair template has been designed, the repair plasmid may be built using simple cloning plasmid backbones (smaller plasmid backbones are preferred). The entire plasmid, including the repair sequence, can be custom‐ordered from most nucleic acid companies or built using traditional plasmid editing techniques such as site‐directed‐mutagenesis (SDM). For FOXA1, we generated two different plasmids, the repair template plasmid and a screening‐PCR positive control plasmid to be used to validate our allele‐specific PCR (ASP; Fig. 3).

To create the repair template plasmid, we used the following primers *FOXA1KITPL‐F*: 5′‐CAAGCTTGCCATGAACAGCATGACTG‐3′ and *FOXA1KITPL‐R*: 5‐CGGATCCCTGAGAAGCAAATGGCTCTG‐3′ to amplify a 1.8kb region surrounding our FOXA1 K295 site from MCF‐7 genomic DNA (Fig. 3). The Phusion High Fidelity DNA Polymerase (NEB Cat # M0530L) was used as per the manufacturer's instructions to amplify the large 1.8 kb fragment. The primers were designed with restriction enzyme site overhangs (BamHI and HindIII) to facilitate cloning into the pGEM‐1 plasmid (Promega; Fig. 3). Of note, the KI site (K295) divides the 1.8 kb genomic fragment into 600 and 1200 bp homology arms, and the design for this particular locus ensures both targeting and screening efficiency [27, 28]. Homology arm length can vary depending on the target locus from < 50 bp on either end, to over 1000 bp [29, 30]. Once cloned into the pGEM1 plasmid, we used the Q5 site‐directed mutagenesis Kit (NEB Cat# E0554S) as per the manufacturer's instructions to introduce the specific nucleic acid substitutions needed to create the repair template sequence shown in Fig. 2. The primers used for site‐directed mutagenesis were *SDM K295Q F*: 5′‐CCGGAGAGCCGCCAGGATCCCTCTGGCGCCTCTAACCC‐3′ and *SDM K295RQ R*: 5′‐TCCGCCTTTAGCCCCAGAGCCCCCGCTTCCGCTCCC‐3′. Once completed, the repair template plasmid was prepped using silica‐membrane‐based kit (Plasmid Miniprep Kit; Qiagen) and sent for Sanger sequencing to confirm proper assembly of the plasmid.

To create the plasmid that will be used as a positive control for the PCR needed for genotyping, the following primers *FOXA1E2TPL‐F*: 5′‐CAAGCTTTTGACAAACTGTGTCACC‐3′ and *FOXA1E2TPL‐R*: 5′‐CGGATCCACCCGTCTGGCTATACTAAC‐3′ were used to amplify 3kb region surrounding the FOXA1 K295 site from MCF‐7 genomic DNA. These primers were also designed with restriction enzyme site overhangs (BamHI and HindIII) to facilitate cloning into the pGEM‐1 plasmid (Promega; Fig. 3). The final step was to introduce the repair template nucleotide substitution sequences into this plasmid. Again, the Q5 Site‐Directed mutagenesis Kit was used along with the same SDM primers described above. This plasmid was intentionally designed to contain more of the endogenous sequence of FOXA1 as well as the repair template sequence to be able to design our ASP (Fig. 3). This is discussed further below. After plasmid prep and Sanger sequencing, we now have a completed allele‐specific screening‐PCR positive control plasmid.

### Transfection and selection of knock‐in cell lines

There are many methods for delivering the sgRNA/Cas9 and the repair template plasmid into cells (i.e., electroporation, viral delivery, or lipid‐based transfections). Each strategy has its own advantages and disadvantages and has been well discussed in these reviews [31, 32]. It is important to choose a transfection method that will be suitable with the intended cell line. We recommend optimizing this procedure with plasmids expressing fluorescent reporters to visualize transfection efficiency and monitor cell toxicity. For MCF‐7 breast cancer cells, we used the lipid‐based Lipofectamine 3000 (Thermo Fisher) transfection reagent due to its low cost and feasibility. If a viral delivery method is preferred, these plasmids would need to be reconstructed accordingly.

To edit FOXA1, we transfected one sgRNA at a time (FOXA1 K295 sgRNA #1) to have the option to perform a second round of transfection with a different sgRNA if some, but not all of the alleles, are successfully edited. For this particular example, using one sgRNA resulted in successful editing of about 54% of the FOXA1 alleles while the remaining alleles resulted in frameshift KO's (thus effectively replacing WT FOXA1 with edited FOXA1). It is possible to successfully edit all alleles of FOXA1 while using only one sgRNA as shown in Table 2, but this is difficult to predict. Therefore, designing several sgRNAs along with a repair template that takes into consideration all sgRNA PAM recognition sites is best. It is possible to transfect all three sgRNAs at the same time, this would increase the on‐target cutting of Cas‐9. However, transfecting three sgRNAs may not necessarily increase the efficiency of HDR, thus there is a chance that even with three sgRNAs, not all of the alleles are successfully edited. If this were to happen, then it would not be possible to perform a second round of transfection because all three PAM sites would have been ablated in the initial round of transfection. In a scenario where all three sgRNAs are transfected at once, any unedited alleles would be repaired using nonhomologous end joining (NHEJ) creating small indels that would result in KO of those alleles and thus ablate the PAM sites (or other critical sequences) that are needed for sgRNA recognition in subsequent transfections. In the case of sequential transfections, the unedited alleles would still contain binding sites for the second or third sgRNA because they are designed slightly upstream or downstream of the first sgRNA used. The successfully edited alleles would remain untouched because the repair template is designed to introduce silent mutations for all three sgRNA PAM sites, preventing these alleles from being targeted in subsequent transfections.

**Table 2:** Quantification of the KI success rate and KI allele frequency estimation for several amino acids targeted in FOXA1.

Amino acid targeted	KI frequency (KI clones/total clones screened)	Average allele frequency (TOPO cloning)	Average allele frequency (RNA‐seq)
K270	10/48	27/28	90%
K270		11/29	41%
K288	13/68	28/28	92%
K288		14/20	55%
K295	7/39	14/30	53%
K295		13/28	28%

The repair template plasmid can be delivered into the cell in a variety of forms. For example, the repair template can be delivered into cells as a circular plasmid, a linearized plasmid, or an isolated template sequence lacking a plasmid backbone; all have been shown to successfully generate KI cell lines. We tested the circular repair template plasmid, the linearized plasmid, a double‐stranded oligonucleotide containing the 1.8 kb repair template sequence, and a single‐stranded 120 bp oligonucleotide centered on the KI site (Fig. 3). After screening at least 35 clones for each type of repair template, we were able to obtain successful KI clones using the circular plasmid but not the other versions of the repair template. Therefore, the circular plasmid is the most efficient method to deliver a repair template sequence into MCF‐7 cells, resulting in a positive KI clone out of every 10–15 clones screened.

For transfection, MCF‐7 cell lines are cultured in DMEM containing 10% FBS and 1% Pen‐Strep and kept in an incubator at 37 °C with 5% CO_2_. Twenty‐four hours prior to transfection, 80 000 cells were seeded in each well of a 12‐well cell culture plate. Optimal seeding density for each cell line should be empirically determined to avoid premature confluency after 4 days of cell culture. The media was replaced with 1 mL of antibiotic‐free culture medium 1 h prior to transfection. Lipofectamine 3000 was used to transfect the cells following the manufactures instructions. Briefly, the transfection complexes contained 1.5 μL of Lipofectamine, 500 ng of pX459 plasmid (FOXA1 K295 sgRNA #1/Cas9), 500 ng of the repair template plasmid, and 2 μL of P3000 in a total volume of 100 μL of Opti‐Mem reduced serum media (Thermo Fisher). After an 8‐h incubation, the cell media/transfection complexes were replaced with fresh antibiotic‐free media to avoid cell toxicity.

The puromycin antibiotic was used for selection of cells that had been successfully transfected. Cells were grown in complete media containing 1 μg·mL^−1^ puromycin for 5–7 days, replacing the culture medium daily. Halfway through selection, the cells were trypsinized and transferred to 60‐mm dishes to spread the remaining cells out and allow for individual clones to grow without colliding into each other. A batch of un‐transfected cells was used to determine when selection with puromycin was complete (i.e., when complete cell death was observed in the control dish).

There are many strategies to pick clones after puromycin selection (serial dilutions, cell lifting, FACS sorting, etc.) [9]. Once visible colonies appeared on the plate, we used glass cloning rings (fixed to the plate with sterile Vaseline) to isolate a particular clone inside the glass ring. Then, trypsin was added to the glass ring to detach the cells and transfer the isolated clone to a separate well within a 24‐well cell culture plate. After expansion of the clone to confluency, the cells were trypsinized and split evenly into two different plates: one plate for genomic DNA extraction and genotyping, and the other plate for maintenance of the clone for continuous culture.

## Results

### Genotyping clonal cell populations using allele‐specific PCR

**Fig. 4:**
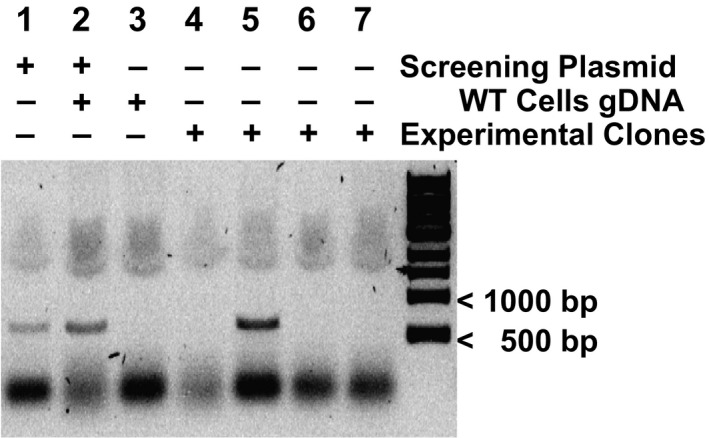
ASP reveals successful FOXA1 KI clones. Agarose gel electrophoresis of ASP results from puromycin‐resistant MCF‐7 clones showing successful editing of FOXA1 at K295 (lane 5). Lanes 1 and 2 are positive controls for the PCR using the ASP positive control plasmid shown in Fig. 3. Genomic DNA from wild‐type MCF‐7 cells was used as a negative control.

We sought to develop a robust genotyping strategy to screen our transfected cell lines for our desired knock‐in allele. To do this, we used ASP. This PCR technique relies on the presence of the nucleotide substitutions specified by the repair template within the endogenous FOXA1 locus. We designed an ASP primer that only anneals to the repair template sequence (Fig. 3). This primer sequence needs to be sufficiently different than the wild‐type DNA sequence (include 3–4 mismatches relative to wild‐type sequence). This will help avoid false positives during PCR screening. The allele‐specific primer was paired with a second primer that is sufficiently upstream of the KI site that it cannot anneal anywhere within the 5′ homology arm sequence included in the repair template (Fig. 3). This generates a specific DNA product that is only possible if there is a successful KI at the endogenous locus (Fig. 4). Importantly, the primer that pairs with the allele‐specific primer must be within the genomic region that is included in the screening‐PCR positive control plasmid but not the repair template plasmid (i.e., the forward primer should not be able to anneal to the repair template plasmid; Fig. 3). This ensures that the ASP screen detects only true KI events and not random integration of the repair template sequence into the host genome. The ASP primers used for FOXA1 K295Q were *K295Q ASP FW*: 5′ ACATGTCCTATGCCAACCCG 3′ and *K295Q ASP RV*: 5′ GCGCCAGAGGGATCCTG 3′.

For genotyping of our clonal cell lines for successful KIs, we used 200ng of purified genomic DNA as the template for the PCR. Genomic DNA was extracted from the individual clones using the Genomic DNA Extraction Kit (Qiagen). As a positive control for the PCR, we mixed 0.4 pg of the screening‐PCR positive control plasmid with 200 ng of wild‐type genomic DNA to mimic physiological concentrations of the expected KI sequence. Based on the plasmid size and polyploid nature of the MCF7 cell line, we determined that 0.4 pg of this plasmid has the same amount of FOXA1 alleles (3–5) as that in 200 ng of genomic DNA [33]. As a negative control, 200 ng of wild‐type genomic DNA was used. Thus, successful ASP would generate a 650 bp PCR product in clones that had successful KI events but not in wild‐type/unedited genomic DNA alone (Fig. 4).

For the PCR, the DNA Taq Polymerase with ThermoPol Buffer from New England Biolabs (NEB, M0267L) was used as per the manufacturer's instructions. The thermocycling conditions were as follows: initial denaturation at 95 °C for 1 min followed by 24 cycles of 95 °C for 15 s, annealing at 68 °C for 15 s, and extension at 68 °C for 42 s. After every 3 cycles, the annealing temperature is decreased by 2 °C until reaching a temperature of 54 °C. Once the temperature reaches 54 °C, five additional cycles are completed (for a total of 29 cycles). The final extension is at 68 °C for 5 min and the sample is held indefinitely at 4 °C. The PCR products were then electrophoresed at 120 volts for 45 min on a 0.8% agarose gel stained with SYBR Safe nucleic acid stain (Thermo Fisher; Fig. 4).

### Knock‐in allele frequency estimation using TOPO cloning, allele‐specific colony PCR, and RNA‐sequencing

**Fig. 5:**
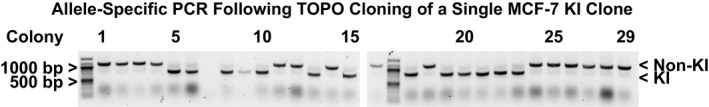
KI allele frequency estimation using TOPO cloning coupled to allele‐specific colony PCR. Allele‐specific colony PCR results from a single KI clonal cell line where 30 bacterial colonies resulting from TOPO cloning of the endogenous FOXA1 target sequence (K295) were screened. Successful KI alleles give a 700‐bp product and wild‐type alleles give a 1000‐bp product. This experiment shows that of the 30 bacterial colonies screened for this one cell line, 13 colonies contained the KI allele, suggesting that about one‐third of the FOXA1 alleles were successfully edited.

For all CRISPR‐based genome engineering experiments, it is important to estimate the allele frequency of successful KI events, especially when working with polypoid cell lines. We used topoisomerase I‐based cloning (TOPO TA Subcloning kit; Thermo Fisher) followed by colony PCR screening to examine the allele composition and KI frequency of FOXA1 in our edited MCF‐7 cells. We reasoned that by amplifying the genomic DNA surrounding the KI site, we would capture all of the different editing events that occurred across the multiple alleles within an individual clonal cell line. By designing primers that are well outside the KI site, the resulting PCR product would contain properly edited alleles together with any remaining indel or wild‐type alleles. Subsequent cloning of the entire heterogeneous PCR product into a TOPO vector (which allows for simple cloning of PCR products without using restriction enzymes), transformation into bacteria, and Sanger sequencing of the resulting bacterial colonies would allow for the estimation of KI alleles in a given cell line based on the ratio of KI alleles to wild‐type/indel alleles found (Fig. 5).

To estimate the KI frequency of FOXA1, genomic DNA was purified from each of clonal cell lines that showed a positive KI result from genotyping. A 1 kb region surrounding the FOXA1 KI site was amplified using the following primers *K295Q ASP FW*: 5′‐ACATGTCCTATGCCAACCCG‐3′ and *F1K SEQ R*: 5′‐GTGCAGCTGGGACTCGTGGG‐3′. The PCR was performed with Taq DNA Polymerase (NEB Cat # M0273S) and, after agarose gel electrophoresis and gel purification, the 1 kb PCR product was ligated into the TOPO 2.1 vector. A total of 5 μL of the ligation reaction was transformed into DH5a competent cells, and 100 μL of the reaction was spread on LB‐ampicillin plates. There are between three and five alleles of FOXA1 in MCF‐7 cells; therefore, we reasoned that screening about 30 bacterial colonies resulting from the topoisomerase‐based cloning (TOPO cloning) reaction would allow us to statistically extrapolate allele frequency (Fig. 5). The colony PCR was designed to contain 3 different primers; two commercially available primers that flank that multiple cloning site of the TOPO cloning vector (M13 and T7) and one allele‐specific primer that would only hybridize if the KI sequence is present. The allele‐specific primer for FOXA1 K295Q is *K295Q ASP RV*: 5′ GCGCCAGAGGGATCCTG 3′. The resulting PCR products would therefore produce a larger 1 kb product if the sequence is wild‐type/indel or a smaller 700‐bp product if the KI sequence is present allowing the allele‐specific primer to anneal (Fig. 5). Thus, this PCR screening method reveals the proportion of FOXA1 KI alleles within a cell line by generating unique PCR products that correlate with KI alleles, in addition to wild‐type/indel alleles (Fig. 5). Sanger sequencing further validated these results and provided insight into the nature of each allele. Alternatively, a 200–300 bp region surrounding the KI site may be amplified and ligated to Illumina‐specific barcodes for sequenced using next‐generation sequencing. The identity and ratio of different FOXA1 alleles could then be extracted using common bioinformatic platforms [34].

**Fig. 6:**
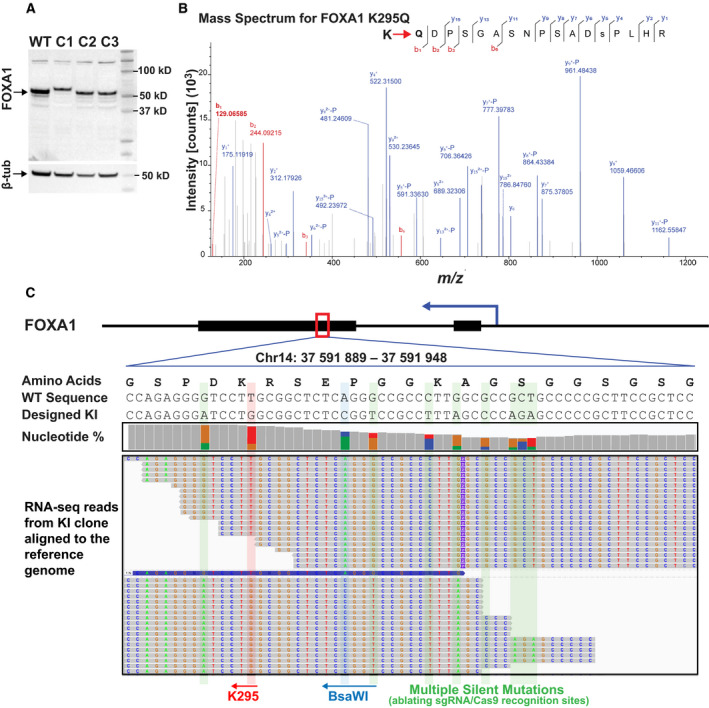
Successful replacement of wild‐type FOXA1 with K295Q KI FOXA1 in MCF‐7 cells confirmed by RNA‐sequencing and mass spectrometry. (A) Western blot of successful FOXA1 KI clones. Western blot showing expression of FOXA1 K295Q KI mutants. One cell line, clone 1, shows a slightly higher molecular weight for FOXA1 potentially hinting at an insertion event. Clones 2 and 3 show appropriate molecular weight for FOXA1 with similar expression levels compared to WT FOXA1. (B) Mass spectrum confirmation of successful K295Q mutagenesis at the endogenous locus of FOXA1. FOXA1 was immunoprecipitated from an MCF‐7 KI clone and subjected to LC‐MS/MS. The fragmentation pattern confirms that lysine (K) at position 295 of FOXA1 was successfully edited to glutamine (Q). (C) Poly‐A enriched RNA‐seq reads from an MCF‐7 KI clone aligned to the FOXA1 reference gene showing edited nucleotides in the mRNA transcripts of FOXA1. This gene is transcribed from the antisense strand and thus the codons are read from right to left. Taken together, these data confirm successful genome editing resulting in appropriate transcription and translation of the intended KI mutations in FOXA1.

As an orthogonal approach to quantify transcription and help estimate allele frequency of the KI mutants, we performed high‐throughput RNA‐sequencing using the Illumina platform. Total RNA from each KI cell line was prepped for Illumina‐based Poly‐A enriched mRNA sequencing as per the manufacturer's instructions (Illumina Stranded mRNA Prep kit). Each RNA‐seq library was sequenced using 150 bp paired‐end sequencing, to a depth of at least 50 million uniquely mapped reads, on an Illumina NextSeq 500 instrument. The RNA‐seq reads were mapped to the human genome reference (GRCh38) using the default parameters in bowtie 1.3.0 and converting the output SAM files into Bam files using SAMtools. The Bam files were then loaded into Integrative Genomics Viewer for visualization of the individual sequencing reads and the proportion of mutated nucleotides within them (Fig. 6C). We were able to confirm the presence of the KI nucleotide changes as well as the silent mutations we designed to ablate the PAM recognition sites. While there were some reads that were seemingly wild‐type, further investigation showed that these transcripts contained indels (insertions or deletions) immediately upstream or downstream of this region which resulted in frameshift knockouts of all wild‐type proteins (Fig 6). It was also possible to estimate proportion of wild‐type to KI sequences by virtue of the percentage of reads containing KI mutations which matched the allele frequency estimations using TOPO cloning. In this case, about 54% of the alleles were successfully edited and the remaining 46% were knockouts. This is reflected in a slight decrease in KI protein expression shown in the western blot (Fig. 6A).

In our attempts to edit several different amino acids in FOXA1, we observed a broad range of KI allele frequency, ranging from 28% to 100% of successfully edited alleles after a single transfection (Table 2). This most likely represents the general numbers that are expected when trying to edit multi‐allelic proteins and can be improved by targeting the unedited alleles with a second transfection using a different sgRNA. Of note, we obtained similar KI allele frequency estimates using the TOPO cloning strategy as compared to the RNA‐seq strategy.

### FOXA1 mutant protein expression analysis by western blotting and mass spectrometry

A potential consequence of editing with the CRISPR‐Cas9 is repair of the resulting double‐strand DNA break without incorporating the intended repair template sequence. Often a random number of nucleotides can be inserted or deleted at the break site and the resulting indel can lead to frameshifts in the coding sequence of the protein. In addition, it is possible that some but not all of the alleles are successfully mutated and the remaining alleles may be wild‐type or knockout. These molecular events can lead to dramatic changes in protein expression or protein size, and thus, western blotting is an important quality control step in ensuring proper expression of the mutated protein (Fig. 6). Of note, it is important to ensure that the desired KI mutations do not ablate the epitope recognized by the antibody chosen for western blot. This is especially true for monoclonal antibodies that only recognize a single epitope. Polyclonal antibodies or combinatorial uses of multiple antibodies (if available) can circumvent this issue. However, the ultimate validation of a successful amino acid substitution is via mass spectrometry. This would provide definitive evidence that the target protein is indeed expressed with the specified amino acid substitution (Fig. 6).

MCF‐7 clones that contained KI sequences based on genotyping were moved forward to western blotting. We followed standard protocols for protein extraction and western blotting to detect FOXA1 in our KI cell lines. Briefly, cells grown in a 10‐cm dish were washed with ice‐cold PBS and collected in 1ml of fresh PBS with protease inhibitors using a cell scraper. Cells were centrifuged at 2400 x ***g*** for 3 min, and the remaining cell pellet was lysed with three times the pellet volume of protein lysis buffer [50 mm Tris pH 8, 0.5 m NaCl, 1% NP‐40, 0.5% sodium deoxycholate, 0.1% SDS, protease inhibitors, and 50 units of Benzonase (Sigma Cat # E8263)] and incubated on ice for 30 min with occasional vortexing. Cell lysates were centrifuged at 12 000 ***g*** for 10 min, and the resulting supernatant was collected for western blot analysis. Protein quantification of the cell lysates was performed using Bradford protein quantification assay (Sigma Aldrich).

Protein lysates were resolved on a 4–12% gradient polyacrylamide gel, transferred to a poly(vinylidene difluoride) membrane, and blotted with a FOXA1 antibody (Abcam, ab23738) diluted 1 : 6000 in 1% BSA. A donkey anti‐rabbit secondary antibody conjugated to HRP was used for visualization (GE Health, NA934‐1ML; Fig. 6).

For mass spectrometry, FOXA1 was first immunoprecipitated from MCF‐7 cell lysates using a FOXA1‐specific antibody (Abcam, ab23738) in order to increase the signal and remove unrelated proteins from the experiment. For immunoprecipitation (IP), protein lysates were generated from 15‐cm dishes as described above and were then diluted 1 : 1 with dilution buffer (20 mm Tris pH8, 150 mm NaCl, 2 mm EDTA, 0.5% Triton X‐100, and protease inhibitors) and subjected to protein quantification. For each IP assay, cell lysates containing 3000 μg of protein extract were further diluted 1 : 10 using dilution buffer and incubated with 5 μg of antibody overnight at 4 °C on a rocking platform. Protein A‐conjugated agarose beads (80 μL, 50% slurry) were washed three times using cold TE buffer and blocked for 2 h using 8 μL of 10 mg·mL^−1^ BSA. The blocked beads were washed two more times with TE buffer and transferred into the IP tube after the overnight incubation with the FOXA1 antibody. With newly added beads, the IP tubes were incubated at 4 °C on a rocking platform for another 2 h. The beads were then pelleted at 400 x ***g***, 4 °C for 1 min, and washed two times with diluted lysis buffer (use the same dilution as for the IP samples). Finally, the beads were washed another time with TE, and the protein sample was eluted in 20 μL 4X SDS loading dye and boiled at 95 °C for 5 min. The boiled sample was centrifugated at 5000 ***g*** for 3 min, and the supernatant was collected for gel electrophoresis and mass spectrometry analysis. The eluted protein was resolved on a 4–12% precast gradient gels from Invitrogen and stained using a standard Coomassie Blue staining protocol. The 50 kD band corresponding to FOXA1 was carefully excised from the gel and submitted to LC‐MS/MS analysis using a ThermoFisher QExactive HF instrument (Fig. 6). As seen in Fig. 6, FOXA1 was successfully identified in the sample and the spectra for the KI mutation (K295 to Q) are shown. Taken together, these data confirm that we have successfully edited the FOXA1 protein sequence by site‐directed mutagenesis of the endogenous loci of FOXA1 in breast cancer cells.

## Discussion

### Precise editing of the endogenous loci of FOXA1 in MCF‐7 cells

Completely replacing a protein coding gene sequence with one that carries targeted mutations in a polyploid cell line is inherently challenging. We have found this to be a labor‐intensive process, extending over 3 months. The goal described in this protocol, to generate KI cell lines capable of producing full‐length FOXA1 mutant protein, demonstrates this point well. We generated 66 clonal cell lines for our FOXA1 K295 KI experiment after antibiotic selection. A total of 12 clones contained the K295Q KI allele and of these 12 positive clones, three were later discarded due to apparent large indels observed via western blot validation of FOXA1 (Fig. 6). From the remaining nine KI clonal cell lines, we selected 4 for TOPO cloning and sequencing. We found small in‐frame deletions within the FOXA1 locus in two of these cell lines making them unusable for our further experiments. The final two cell lines contained our desired FOXA1 KI alleles along with nonfunctional indel alleles (knockouts), thus successfully replacing all wild‐type versions of FOXA1 in these cells, making them ideal for downstream experiments. Based on our allele frequency estimation, we obtained 2–3 successful KI alleles of FOXA1 in our MCF‐7 cell lines which contain about 3–5 alleles total of FOXA1 (Fig. 6) [33]. From the RNA‐seq and TOPO cloning allele frequency data of these selected KI cell lines, we found that 28–100% mature mRNA in these cell lines carry KI mutations and also found the presence of several KO alleles (Table 2). Our results suggest that the KI frequency is limited by the efficiency of the HDR pathway when competing with the more facile NHEJ pathway. This scenario illustrates the success rate in generating suitable cell lines and suggests that one should aim to generate 2 or 3 times more total cell lines than actually needed. Of note, our design of the repair template used for homologous recombination allows for a second round of KI targeting and screening, which will eventually lead to complete replacement of all endogenous alleles with KI alleles.

### Determining HDR competency of targeted cell lines

Most actively proliferating cell lines are suitable for CRISPR/Cas9 directed site‐specific mutagenesis. Some cell lines, however, may have inherent defects in the HDR pathway and it is important to consult existing literature for evidence of successful genome editing in the cell line of interest [27]. CCLE (Broad Institute Cancer Cell Line Encyclopedia, https://portals.broadinstitute.org/ccle) is a useful resource to survey potential cell lines for defects in critical components of the HDR machinery [35]. Finally, recombination activity tests may be attempted in a desired cell line for further assurance [36, 37].

### Considerations in designing targeting and repair template sequences

To reduce off‐target effects, it is beneficial to choose targeting sequences that display higher specificity even if there is some compromise in efficiency. If necessary, the most likely off‐target sites (as predicted by CRISPOR) can be surveyed and examined by Sanger sequencing to detect unintended alterations at these other loci.

Previous work has indicated that the DNA sequence at the Cas9 cleavage site is more likely to be repaired by an exogenous repair template than adjacent sequences [38, 39]. When designing the repair template, the silent mutations (preventing repeated cleavage by sgRNA/Cas9, or to introduce restriction sites) are best introduced between the gRNA targeting sequence and the genomic editing site. These silent mutations should be clustered together, rather than dispersed, to ensure incorporation of all of the intended substitutions. Moreover, this protocol can be easily adjusted to work with Cas9 Nickase, which requires two nearby sgRNA targeting sequences for proper recognition and cleavage, which will increase on‐target specificity.

## Conclusions

There are a handful of strategic paths that may be taken to further increase KI efficiency using CRISPR‐Cas9 including second round of CRISPR‐Cas9 KI editing using different sgRNA. Alternatively, designing a dual antibiotic selection strategy where two selectable markers are separately added to the same repair template would facilitate dual antibiotic selection for cells that integrated KI cassettes into all alleles [40]. Another strategy for increasing efficiency could be chemical inhibition of the NHEJ repair pathway thus forcing the cells to engage in HDR [40, 41, 42]. Finally, CRISPR‐trap, an alternative genome editing strategy, could be adopted to introduce KI proteins while avoiding frameshift truncations [43, 44]. The downside with this strategy, however, is the KI transcripts will lose their endogenous 3′ UTR and corresponding native gene regulation [43].

As more CRISPR editing tools emerge, our ability to edit endogenous loci of polypoid cell lines will continue to increase. For example, the recent development of CRISPR‐based Prime Editing can directly write new genetic information into a specified DNA site using a catalytically impaired Cas9 endonuclease fused to an engineered reverse transcriptase. Prime editing is programmed with a sgRNA that both specify the target site and encodes the desired edit, and thus has the potential to readily modify the majority of endogenous proteins in polyploid cancer cell lines [45]. The strategies described herein, sgRNA design, repair template design, genotype screening strategies, and allele frequency estimation, would still apply and greatly facilitate the use of these other CRISPR technologies such as prime editing.

## Conflict of interest

The authors declare no conflict of interest.

## Author contributions

HLF, SL, and JPG conceived the project and developed the methodology. SL and CAT performed the experiments with input from HLF and JPG. All authors wrote and approved the final manuscript.

## Data Availability

The materials developed and the datasets analyzed during the current study are available from the corresponding author upon request.
